# Bone regeneration using PRF, PRP and β-TCP in bone defects

**DOI:** 10.6026/973206300210793

**Published:** 2025-04-30

**Authors:** Vipin Arora, Ankur Joshi, Nishita Anthwal, Sameer Gupta, Ashtha Arya, Mahima Paul

**Affiliations:** 1 Department of Restorative Dental Sciences, Taif University, Kingdom of Saudi Arabia - 21944; 2 Department of Dentistry, Government Medical College, Haridwar - 249401, Uttarakhand, India; 3 Department of Oral Pathology and Microbiology, UDMRI, Dehradun, Uttarakhand - 248140, India; 4 Department of Oral and Maxillofacial Surgery, Inderprastha Dental College and Hospital, Sahibabad, Ghaziabad, Uttar Pradesh, - 201010, India; 5 Department of Conservative Dentistry and Endodontics, SGT Dental College, Hospital and Research Institute, SGT University, Gurugram, Haryana - 122505, India; 6 Department of Oral Medicine and Radiology, ⁠Kalinga Institute of Dental Sciences, KIIT Deemed To Be University, Patia, Bhubaneswar, India

**Keywords:** Bone regeneration, platelet-rich fibrin (PRF), platelet-rich plasma (PRP), beta-tricalcium phosphate (β-TCP), bone defects, osteogenesis

## Abstract

Bone regeneration using platelet-rich fibrin (PRF), platelet-rich plasma (PRP) and beta-tricalcium phosphate (β-TCP) in bone defects is of interest. PRF achieved the maximum bone fill (85%) during the 12 weeks assessment period while PRP and β-TCP reached 75% and 65% bone fill, respectively. Trabecular bone density proved superior in defects treated with PRF according to the histological findings (p < 0.05). The prolonged availability of growth factors from PRF resulted in better bone tissue formation. The biomaterial PRF demonstrates strong potential to become an effective solution for efficient bone healing in clinical medicine.

## Background:

The biological process of bone regeneration consists of integrated actions between osteogenic cells while triggering growth factors and extracellular matrix components. PRP + β-TCP and PRF + β-TCP combinations for regenerative healing of chronic periapical lesions, shows promising results quantitatively through CBCT over six months to one year [1]. The medical community adopts platelet concentrates including platelet-rich fibrin (PRF) and platelet-rich plasma (PRP) due to their natural origin as well as their superior capability to support bone healing by gradually releasing growth factors [2, 3]. Regenerative endodontic procedures incorporating biomaterials have shown promising healing outcomes when assessed using advanced imaging modalities like CBCT [4]. The use of bone grafts and guided tissue regeneration techniques has demonstrated efficacy in enhancing periodontal healing and bone regeneration [5]. PRP stands as a first-generation platelet concentrate that contains a high platelet concentration and growth factors but needs external activation for its regenerative capacity to become effective [6]. Studies about the comparing efficiency of PRF and PRP in bone regeneration continue to be investigated in current research [7]. Platelet-rich plasma, platelet-rich fibrin and platelet pellet can be effective alternatives to blood clot as scaffolds offering similar healing outcomes [8]. The combination of platelet concentrates with bone graft materials enhances periapical bone regeneration in endodontic surgeries [9]. Growth factors released from platelet-derived biomaterials play a critical role in modulating inflammation and promoting tissue regeneration [10]. Therefore, it is of interest to assess PRF performance relative to PRP and β-TCP within induced bone defects for bone healing using radiographic and histological along with micro-CT techniques.

## Materials and Methods:

## Study design and animal model:

This scientific research included fifteen living male rabbits weighing between 2.5 to 3.0 kg for laboratory testing. Thirty defects distributed among three groups consisted of Group A with PRF and Group B with PRP while Group C received β-TCP for analysis. The study obtained approval from the Institutional Animal Ethics Committee to use established guidelines for laboratory animal care.

## Surgical procedure and defect creation:

Ketamine at a dosage of 35 mg/kg combined with xylazine at a dosage of 5 mg/kg served to anesthetize the animals through intramuscular injection. The surgeon began by confirming full anesthetic effect before performing a central mouth opening procedure on the mandible bone while paying strict attention to bone tissue exposure. A sterilized surgical drill under low-speed ran saline irrigation created the standardized bone defect which measured 5 mm in diameter and extended 3 millimeters into the bone.

## Preparation of PRF, PRP and β-TCP:

The researcher drew 10 mL whole blood from the vein then performed 3000 rpm centrifugation for ten minutes without anticoagulants to prepare PRF. A mammified PRF membrane structure was obtained by collecting its clot from the red blood cell layer followed by compression. The PRP preparation involved obtaining 10 mL blood from tubes with anticoagulants before performing 1500 rpm centrifugation for ten minutes. Activation of PRP involved centrifuging a portion of plasma which had been separated from platelet-rich platelets again at 2500 rpm for ten minutes. A physician activated the PRP solution through calcium chloride treatment before clinical application. Several steps were followed for β-TCP preparation including commercial sterilization of β-TCP granules followed by hydrating them with saline solution before surgical implantation.

## Experimental groups and implantation:

The researchers used freshly obtained PRF to fill the surgical site in group A. Laboratory Group B received the application of PRP gel for filling the treatment area. The researchers filled the defect with β-TCP granules during Group C procedures. A postoperative antibiotic treatment of enrofloxacin 5 mg/kg combined with the administration of meloxicam 0.2 mg/kg was given for three consecutive days after using resorbable sutures to close the surgical area.

## Postoperative assessment and data collection:

Particle analysis for bone healing occurred at 4 weeks 8 weeks along with 12 weeks using three different testing procedures.

[1] A digital X-ray system took images during every measurement interval for evaluation of bone density and defect recovery.

[2] The micro-CT analysis generated three-dimensional pictures of the affected site allowing researchers to evaluate bone volume percentage along with trabecular architectural assessment.

[3] At each time point postoperatively the animals received euthanasia followed by extraction of mandibles for histological assessment using decalcification techniques and subsequent staining with hematoxylin and eosin for histomorphometric measurement. The research evaluated new bone formation together with trabecular thickness and osteoid presence as key parameters.

## Statistical analysis:

The data analysis took place through SPSS version 25.0, USA). A one-way ANOVA method and Tukey's post-hoc test evaluated the mean bone regeneration percentages between the three study groups. The research considered a p-value less than 0.05 as the threshold for statistical significance.

## Results:

PRF together with PRP and β-TCP went through bone regeneration testing using radiographic and micro-CT methods and histological examinations at weeks 4, 8, and 12. The analyzed data from the three groups produced distinct results for bone fill percentages and trabecular thickness and newly generated bone volume measurements.

## Radiographic evaluation:

The bone fill analysis of three groups through radiography revealed progressive development of new bone tissue and PRF obtained maximum bone recovery at each assessment time. The bone regeneration within PRF-treated defects reached 35% at 4 weeks but both PRP and β-TCP yielded lower results of 30% and 25%. At 8 weeks post-surgery PRF-treated sites achieved 65% bone regeneration whereas both the PRP group reached 55% and the β-TCP group achieved only 45%. Results at week 12 showed that PRF achieved the greatest bone fill percentage of 85% which exceeded PRP at 75% and β-TCP at 65% (Table 1).

## Micro-CT analysis:

Micro-computed tomography (micro-CT) evaluation provided a quantitative assessment of bone volume percentage and trabecular thickness. At 4 weeks, the mean trabecular thickness was 0.45 ± 0.05 mm in the PRF group, 0.40 ± 0.04 mm in PRP, and 0.35 ± 0.03 mm in β-TCP. By 12 weeks, PRF-treated defects had a significantly higher trabecular thickness (0.85 ± 0.06 mm), compared to PRP (0.78 ± 0.05 mm) and β-TCP (0.65 ± 0.04 mm) (Table 2).

## Histological and histo-morphometric evaluation:

Histological examination revealed increased osteoid formation and trabecular bone development in the PRF group compared to PRP and β-TCP. At 4 weeks, PRF-treated defects showed 40% new bone formation, whereas PRP and β-TCP had 35% and 28%, respectively. By 12 weeks, the PRF group exhibited 88% bone formation, PRP 78%, and β-TCP 68% (Table 3).

## Statistical analysis:

The one-way ANOVA test revealed a statistically significant difference (p < 0.05) among the three groups in terms of bone fill percentage, trabecular thickness, and new bone formation. Tukey's post-hoc analysis indicated that PRF performed significantly better than PRP and β-TCP at all-time intervals (p < 0.05). Overall, PRF demonstrated superior bone regeneration capabilities compared to PRP and β-TCP, as evidenced by radiographic, micro-CT, and histological findings.

## Discussion:

The healing process of bone regeneration plays a vital role in surgical treatment procedures of dentistry and orthopedics where various biomaterials act as healing-enhancement tools. This research evaluated the bone regenerative properties of PRF alongside PRP and β-TCP in artificially made bone cavities. The research showed PRF generated better bone tissue than PRP and β-TCP based on X-ray results combined with micro-CT and histological analysis. Because they contain high factors including PDGF and TGF-β and VEGF that stimulates osteoblast growth and promotes new blood vessel formation [1, 2], PRF and PRP serve as autologous platelet concentrates. The second-generation platelet concentrate PRF combines fibrin network scaffolding with sustained growth factor release mechanism which creates better bone formation conditions [3, 4].

PRF-treated defects demonstrated superior results in both bone tissue regeneration metrics of fill and trabecular thickness than those treated with PRP according to confirmed previous research findings [5]. The platelet levels in PRP are higher than other platelet concentrates yet the lack of a fibrin network triggers swift growth factor dissolution which reduces its overall operational period to six months [6]. The osteogenic activity of PRP proved to be lower than PRF while testing their bone healing capacities [7, 8] as reported earlier studies show. Research results establish that PRP speeds up initial healing responses but the sustained regenerative properties belong to PRF [9]. Research suggests β-TCP stands among the most chosen synthetic graft materials because it helps bone tissues grow while providing structural support for tissue development [10]. The absence of osteo-inductive properties in β-TCP hinders its ability to regenerate as effectively as platelet concentrates [11]. The β-TCP-treated defects showed delayed and reduced bone formation compared to PRF as well as PRP according to existing research reports [12]. β-TCP promotes new bone growth but needs to be combined with autologous growth factors and stem cells to match the effects of biological alternatives [13]. The bone filling percentage and trabecular thickness as well as new bone formation analysis showed peak results with PRF comparison to PRP and β-TCP. The observed findings confirm that PRF stands as a superior and clinical-ready solution for bone tissue regeneration especially within periodontal and maxillofacial treatments [14]. Growth factor delivery via PRF provides extended osteogenic effects that make it an ideal material for practical clinical procedures [15].

## Conclusion:

PRF showed the optimal regenerative outcome. However, healthcare professionals must balance it with availability requirements and processing time duration together with patient-by-patient reactions. The combination of these treatments provides relevant medical options that serve different purposes in bone healing scenarios.

## Figures and Tables

**Table 1:** Bone fills percentage at different time intervals

**Time Interval**	**PRF (%)**	**PRP (%)**	**β-TCP (%)**
4 weeks	35	30	25
8 weeks	65	55	45
12 weeks	85	75	65
Comparison of bone fills percentage among the three groups over time.

**Table 2:** Micro-CT assessment of trabecular thickness (mm)

**Time Interval**	**PRF (mm)**	**PRP (mm)**	**β-TCP (mm)**
4 weeks	0.45 ± 0.05	0.40 ± 0.04	0.35 ± 0.03
8 weeks	0.68 ± 0.05	0.60 ± 0.04	0.50 ± 0.03
12 weeks	0.85 ± 0.06	0.78 ± 0.05	0.65 ± 0.04
Trabecular thickness measurements obtained from micro-CT analysis.

**Table 3:** Histological assessment of new bone formation (%)

**Time Interval**	**PRF (%)**	**PRP (%)**	**β-TCP (%)**
4 weeks	40	35	28
8 weeks	70	62	50
12 weeks	88	78	68
Comparison of new bone formation percentage based on histological assessment.
